# Biodiversity of Jinggangshan Mountain: The Importance of Topography and Geographical Location in Supporting Higher Biodiversity

**DOI:** 10.1371/journal.pone.0120208

**Published:** 2015-03-12

**Authors:** Ting Zhou, Bao-Ming Chen, Gang Liu, Fang-Fang Huang, Jin-Gang Liu, Wen-Bo Liao, Ying-Yong Wang, Si-Jie Ren, Chun-Quan Chen, Shao-Lin Peng

**Affiliations:** 1 State Key Laboratory of Biocontrol and Guangdong Key Laboratory of Plant Resources, School of Life Sciences, Sun Yat-sen University, Guangzhou, 51027, China; 2 Administration Bureau of Jinggangshan, Ji’an 343600, China; Wuhan Botanical Garden, Chinese Academy of Sciences, Wuhan, China, CHINA

## Abstract

Diversity is mainly determined by climate and environment. In addition, topography is a complex factor, and the relationship between topography and biodiversity is still poorly understood. To understand the role of topography, i.e., altitude and slope, in biodiversity, we selected Jinggangshan Mountain (JGM), an area with unique topography, as the study area. We surveyed plant and animal species richness of JGM and compared the biodiversity and the main geographic characteristics of JGM with the adjacent 4 mountains. Gleason’s richness index was calculated to assess the diversity of species. In total, 2958 spermatophyte species, 418 bryophyte species, 355 pteridophyte species and 493 species of vertebrate animals were recorded in this survey. In general, the JGM biodiversity was higher than that of the adjacent mountains. Regarding topographic characteristics, 77% of JGM’s area was in the mid-altitude region and approximately 40% of JGM’s area was in the 10°–20° slope range, which may support more vegetation types in JGM area and make it a biodiversity hotspot. It should be noted that although the impact of topography on biodiversity was substantial, climate is still a more general factor driving the formation and maintenance of higher biodiversity. Topographic conditions can create microclimates, and both climatic and topographic conditions contribute to the formation of high biodiversity in JGM.

## Introduction

Drivers of biodiversity have long been of interest in ecology [1,2,3,4,5]. Latitude, longitude and altitude are well known to be the main factors shaping the spatial patterns of species diversity at large scales [6,7,8,9,10].

Many studies have discussed the roles of climate and land cover in determining individual species distributions [11,12,13]. Kerr et al. (2001) studied the roles of energy and habitat heterogeneity in determining biodiversity [14]. Complex habitats can provide more available niches, and therefore high species richness can be found in these habitats [15]. Species richness has been shown to be determined by climate, topography and vegetation structure, and vegetation complexity should be taken into account in habitat management and biological conservation [16]. Gutiérrez Illán et al. (2010) demonstrated strong topoclimatic effects on butterfly diversity in a mountain range. Climate factors related to elevation appear to be the most important predictors of butterfly species richness on a fine scale [17].

Topography plays an important role in the distribution of vegetation and biodiversity [17,18,19,20,21]. Particularly, at local scales, topographic variability is critical in determining plant species distributions [22,23]. In mountain areas, the vegetation structure and species composition of forests characteristically change along altitudinal gradients. It has been well documented that there is an overall decrease in tree species richness, forest height and aboveground primary productivity with increasing elevation [24,25] and that there is relationship between local species distribution and altitude [26,27,28,29,30]. Topography is a complex factor and relates to hydrology, nutrient dispersion, soil structure and microclimate, which are difficult to disentangle. The relationship between topography and biodiversity is still poorly understood [31,32]. A unique geographic location may play a role in maintaining biodiversity in addition to the microclimate and environment. For example, a junction center could be a biodiversity hotspot due to its geographic advantage over adjacent patches.

Jinggangshan Mountain (JGM) is located in the southwest of Jiangxi Province, China, where the Jinggangshan National Nature Reserve (JNNR) is located. The mountain runs in a north-south direction and is surrounded by 4 mountains, including the north-south-oriented Luoxiao Mountain (LXM) to the north, the northeast-southwest-oriented Wuling Mountain (WLM) to the west, the northeast-southwest-oriented Wuyi Mountain (WYM) to the east and the west-east-oriented Nanling Mountain (NLM) to the south. The four mountains crisscross one another and JGM is located in the center of them.

JGM is an important hotspot of biodiversity in southern China, where JNNR is an important national nature reserve to protect biological resources in China [33]. However, only a few studies have surveyed the local biodiversity (e.g., reptiles, butterfly, birds) [33,34,35,36]. To better understand the biodiversity within this reserve, we conducted a systematic biodiversity survey concerning plant (spermatophyte, bryophyte and pteridophyte) and animal (hexapod, fish, amphibian and reptilian, avian and mammalian) species. In addition to biodiversity, some researchers have studied topographic characteristics and formation mechanisms in JGM [37]. However, they did not link this information with the biodiversity in this region. We predict that the unique geographic location of JGM may play an important role in maintaining biodiversity. In this study we explored the potential roles of topography, i.e., altitude and slope, on the biodiversity in JGM by conducting a thorough survey in this area.

## Materials and Methods

### Ethics Statement

Field studies were approved by the Nature Reserve Administration Bureau of Jinggangshan for species survey in JGM. Field studies did not involve endangered or protected species. For vertebrate studies, full details of collection and sampling methods are mentioned below (Animal survey). The work was approved by the Jiangxi Provincial Bureau of Forestry and the Nature Reserve Administration Bureau of Jinggangshan, which are equivalent with the Institutional Animal Care and Use Committee (IACUC).

### Study site

**Fig 1:**
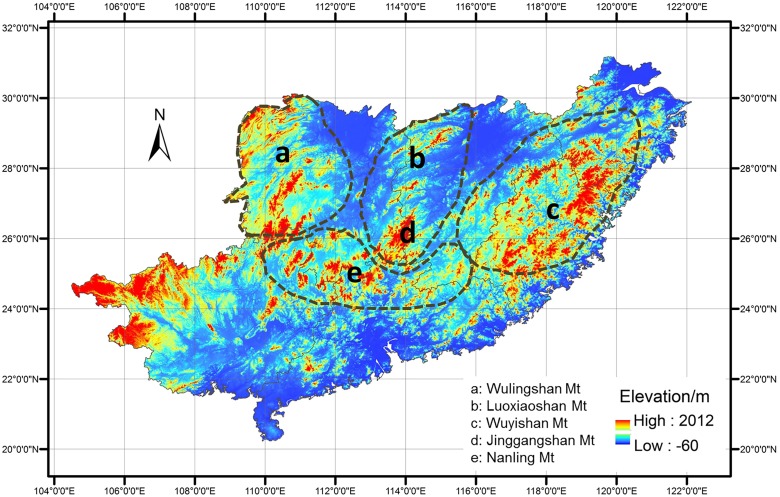
Geographic position of Jinggangshan Mountain formed by convergence of four mountains to one point.

JGM is located in the southwest of Jiangxi Province, China (26°28′39″–26°40′03″ N, 114°04′05″–114°16′38″ E), covering an area of 261.4 km^2^. The region lies in the mid-subtropical monsoon zone with mean annual precipitation of 1889.8 mm and relative humidity of 85%. The mean annual temperature is 14.2°C, with average temperatures of 23.9°C and 3.4°C in the hottest (July) and coldest (January) months, respectively. There is wide variation in microtopography on JGM, including mountain ridges, cliffs, deep canyons, basins and other features. The diverse microtopographical systems and tremendous elevation drop (1900 m) leads to differential distributions of heat and moisture over space and time, contributing to the formation of various soil and vegetation types. JGM is adjacent to three mountains (WLM to the west, WYM to the east and NLM to the south) (Fig. 1), which contribute to the unique topography of JGM. Wuyi Mountain (to the east of JGM) partly blocks the eastern marine monsoon, and Wuling Mountain (to the west of JGM) partly blocks the northwestern cold air, forming a relatively enclosed region in JGM.

### Species survey

The plant survey was conducted mainly in JNNR. In addition, Jiangxi Nanfengmian Provincial Nature Reserve and Jiangxi Qixiling Provincial Nature Reserve were included, both of which are located in JGM. The animal survey was confined to JNNR. The term JGM was used below to represent JNNR and the surrounding nature reserves. Since the nature reserve suffers the least disturbance across JGM, thus supporting the highest biodiversity, we believed that species survey in this area is representative of the whole JGM.

### Plant survey

Spermatophyte survey: Dozens of scientific investigations were conducted in JGM from 2009 to 2011. The investigation covered all vegetation types. Field surveys were conducted in typical sample plots of the main vegetation types, and the rest were described. Each plot contained 2–20 quadrats of 10×10 m^2^ according to varying community characteristics. In total, 550 quadrats were established and investigated. In each quadrat, we measured the height and diameter at breast height (DBH) of all the trees and recorded the coverage of herbage. More than 6500 plant specimens were collected and identified during the investigations. We also referred to plant specimens conserved in JNNR Herbarium. A list of spermatophytes in the JGM was compiled based on the survey results.

Bryophyte and pteridophyte survey: Specimens of moss and fern were collected during the spermatophyte survey. Special investigations were also conducted to identify the diversity, distribution and population size of moss and fern species. A list of bryophytes and a list of pteridophytes in the JGM were compiled from the surveys.

### Animal survey

Hexapod survey: Eight field investigations were conducted in JGM from October 2010 to October 2011. Approximately 25,000 specimens were captured and 800 pictures were taken. The main capture methods included sweeping, scooping, light trapping, bark stripping and litter collection. A list of hexapods in the JGM was compiled based on the collected specimens.

Fish survey: In total, 29 sampling points covering the main water body were set in the JNNR. With the assistance of local staff and fishermen, fish were captured once a month in a 100-m^2^ water area from April 2011 to July 2011. A list of fish in the JGM was compiled based on the survey.

Amphibian and reptilian survey: 12 field investigations were conducted at 30 sites in JNNR from September 2010 to July 2012. The cumulative time period for these surveys was 140 days. No more than 4 specimens were captured in each site. A list of amphibians and a list of reptiles in the JGM were compiled based on the survey data.

Avian survey: Field investigations were conducted monthly in the JGM from September 2010 to November 2011. Each investigation lasted 10 days. A belt transect method was used to cover all the habitat types in the JGM. Birds were observed through a binocular telescope and identified by their figure, sound and flying posture. Pictures were taken, and sounds were recorded whenever possible. The survey results were summarized in a list of avian species in the JGM.

Mammalian survey: Mammals were surveyed from September 2010 to February 2012. The same belt transect method was used for the avian, amphibian and reptile surveys. In addition, steel traps and net catchers were used to capture rodent and chiropteran species, respectively. We also consulted local villagers and referred to the specimen collection of the JNNR Herbarium. The survey results were summarized in a list of mammals in the JGM.

Animals were released after identification. All the voucher specimens collected from JGM were deposited in the Biological Museum of Sun Yat-sen University.

### Data collection

The nature reserves located in WLM, LXM, NLM and WYM were selected as the control areas. Species data of those nature reserves were collected from the published literature and processed for comparison with JGM. Jiangxi Guanshan National Nature Reserve (28°30′–28°40′ N, 114°29′–114°45′ E) is located in the north of LXM. Hunan Wulingyuan (29°18′–29°25′N, 110°27′–110°39′ E) and Nature Reserve (30°02′–30°08′N, 110°29′–110°40′ E) were located in WLM. Fujian Wuyishan National (27°32′–27°55′N, 117°24′–118°02′ E) and Guangdong Nanling National (24°37′–24°57′N, 112°30′–113°04′ E) were located in WYM and NLM, respectively.

### Topographical analysis

On mountains, the peak in species richness is predicted to occur at mid-altitudes, where a unimodal water-availability gradient reaches a maximum while temperatures are still relatively high [38,39]. To analyze topographical differences among the mountains, we calculated the altitude and slope of the 5 nature reserves. First, searches were conducted in Google Earth based on geographical coordinates, and the maps were exported as vector fields. Second, we extracted regions of interest from the Shuttle Radar Topographic Mission Digital Elevation Models (SRTM DEM) data and SRTM SLOPE data using vector ranges as masks in the “spatial analyst” toolbox in ArcGIS 10.0. Third, altitude data and slope data corresponding to each mountain were classified at intervals of 200 m (for altitude) or 10° (for slope) in the density slicing tool in the Environment for Visualizing Images 4.8 (ENVI). Finally, using statistical tools in ENVI 4.8, the percentage of each class was calculated by dividing the total area of the mountain by the class area.

### Overlapping species collection

Based on the species survey in JGM and the data collected from the other 4 mountains, we identified some species with overlapping distributions in JGM and one of the adjacent mountains but do not exist on the other 3 mountains.

### Data Analysis

The Gleason richness index was calculated to assess the diversity of species. The index indicates richness based on species density to eliminate the effect of area on species number. The Gleason richness index was calculated as follows [40]:

D=S/LnA

where *S* = species number of a given community, and *A* = area of a given community.

## Results

### The biodiversity of spermatophytes

**Fig 2:**
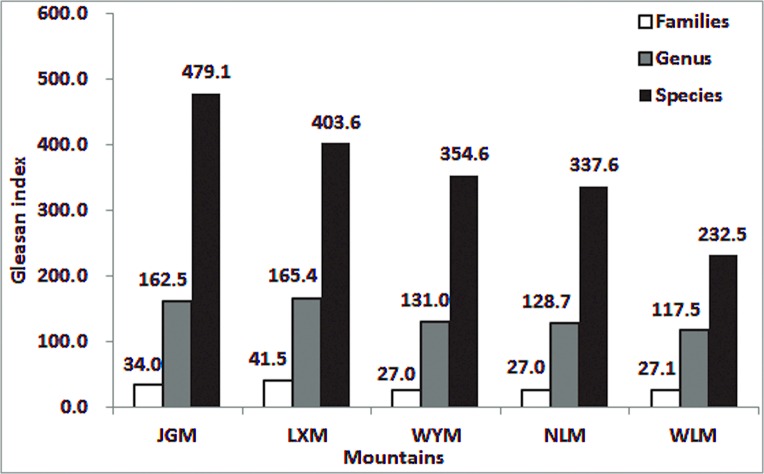
Comparison of the Gleason index of seed plant species, genera and families of Jinggangshan Mountain (JGM) and surrounding mountains. LXM, Luoxiao Mountain; WYM, Wuyi Mountain; NLM, Nanling Mountain; WLM, Wuling Mountain.

In total, 2958 spermatophyte species belonging to 1003 genera and 210 families were found on JGM. The number of seed plant species and genera on JGM was the greatest among the studied mountains. Species that are endemic in China were abundant on JGM. According to “China Flora” (2005) [41], there are 16,864 endemic species in China, including 36% of the seed plants in China. Of these endemic seed species, 1146 species (423 genera and 131 families) were found on JGM. The genera of seed plants on JGM accounted for 42.17% of the genera endemic to China. This percentage was higher than the percentages from WLM (9.7%, 72 genera), WYM (3.3%, 27 genera) and NLM (6.4%, 39 genera) [42,43]. The Gleason index for seed plant families, genera, and species on JGM was 34.0, 162.5 and 479.1, respectively. These index values were higher than the values from surrounding mountains (Fig. 2).

### The biodiversity of bryophytes

In total, 418 bryophyte species (183 genera, 67 families) were recorded in this survey, including 8 subspecies and 6 variants. The number and Gleason index of species were relatively high (Table 1).

**Table 1:** Comparison of the abundance of bryophyte species, genera and families of JGM and surrounding mountains.

	No. of families	No. of genera	No. of species	Area /km^2^	Gleason index		
					families	genera	species
JGM	67	183	418	480	10.9	29.6	67.7
LXM	61	136	238	115	12.9	28.7	50.2
WYM	74	191	358	565.3	11.7	30.1	56.5
NLM	53	115	209	563	8.4	18.2	33.0
WLM	33	90	192	409.7	5.5	15.0	31.9

JGM, Jinggangshan Mountain; LXM, Luoxiao Mountain, WYM, Wuyi Mountain; NLM, Nanling Mountain; WLM, Wuling Mountain. The data for WLM, LXM, WYM and NLM come from surveys in Houhe Natural Reserve [44], Guanshan Natural Reserve [45], Wuyishan Area, Fujian Province [46] and Nanling National Natural Reserve [47], respectively.

### The biodiversity of pteridophytes

In total, 355 pteridophyte species (104 genera, 46 families) were found on JGM. The most abundant species belonged to genera *Selaginella*, *Pteris*, *Arachniodes*, *Dryopteris*, *Polystichum* and *Asplenium*. The Gleason index for JGM was much higher than that of WLM, WYM, LXM, and NLM (Table 2).

**Table 2:** Comparison of the biodiversity of pteridophyte species, genera and families of JGM and surrounding mountains.

	No. of families	No. of genera	No. of species	Area /km^2^	Gleason index		
					families	genera	species
JGM	46	104	355	480	7.5	16.8	57.5
LXM	36	79	191	115	7.6	16.6	40.3
WYM	40	85	280	565.3	6.3	13.4	44.2
NLM	43	94	188	563	6.8	14.8	29.7
WLM	38	86	256	552	6.0	13.6	40.5

JGM, Jinggangshan Mountain; LXM, Luoxiao Mountain, WYM, Wuyi Mountain; NLM, Nanling Mountain; WLM, Wuling Mountain. The data for WLM, LXM, WYM, and NLM come from surveys in Wulingyuan Natural Reserve [48], Guanshan Natural Reserve [45], Wuyishan Area, Fujian Province [46] and Nanling National Natural Reserve [49], respectively.

### The biodiversity of protected and endangered plant species

In total, 201 species of protected and endangered plants (111 genera, 46 families) were found on JGM. Among these plants, 134 species (90 genera, 35 families) were angiosperms, 23 species (18 genera, 8 families) were gymnosperms and the remaining 4 species (3 genera, 3 families) were pteridophytes. Among these records, 51 types of protected and endangered plants were recorded in the IUCN Red List of Threatened Species [50], 161 wild kinds were recorded on the China Species Red List [51], 49 types of key protected plants were recorded on “The List of National Key Protected Wild Plants” [52], and five types of the key protected plants have been listed as first-class protected species, e.g., *Ginkgo biloba*, *Abies beshanzuensis var*. *ziyuanensis*, *Taxus wallichiana var*. *mairei*, *Bretschneidera sinensis and Dendrobium hancockii*.

Both the number and the Gleason index of protected and endangered plants of JGM were greater than the values from the four surrounding mountains (Table 3).

**Table 3:** Comparison of the biodiversity of protected and endangered plants of JGM and surrounding mountains.

	National Key Protected Species		IUCN Red List of Threatened Species				China Species Red List				Gleason index
	I	II	CR	EN	VU	NT	CR	EN	VU	NT	
JGM	5	44	1	5	15	14	5	14	74	66	25.75
LXM	3	18	-	-	-	-	0	3	11	14	5.9
WYM	4	23	1	5	14	20	1	5	64	42	17.67
NLM	-	-	-	-	-	-	0	2	20	14	5.68
WLM	8	28	-	-	-	-	-	-	-	-	5.7

JGM, Jinggangshan Mountain; LXM, Luoxiao Mountain, WYM, Wuyi Mountain; NLM, Nanling Mountain; WLM, Wuling Mountain. I, first class national key protected species; II, second class national key protected species; CR, critical species; EN, endangered species; VU, vulnerable species; NT, near threatened species. The data for LXM, WYM, NLM and WLM came from surveys in Guanshan Natural Reserve [45], Wuyishan Area, Fujian Province [46], Nanling National Natural Reserve [53] and Wulingyuan Natural Reserve [54], respectively.

### The biodiversity of vertebrates

In this research, 493 species of vertebrate animals (109 families, 34 orders, 5 classes) were found, including 38 species of fishes (12 families, 4 orders), 40 species of amphibians (9 families, 2 orders), 64 species of reptiles (12 families, 3 orders), 287 species of birds (56 families, 17 orders) and 64 species of mammals (20 families, 8 orders).

**Fig 3:**
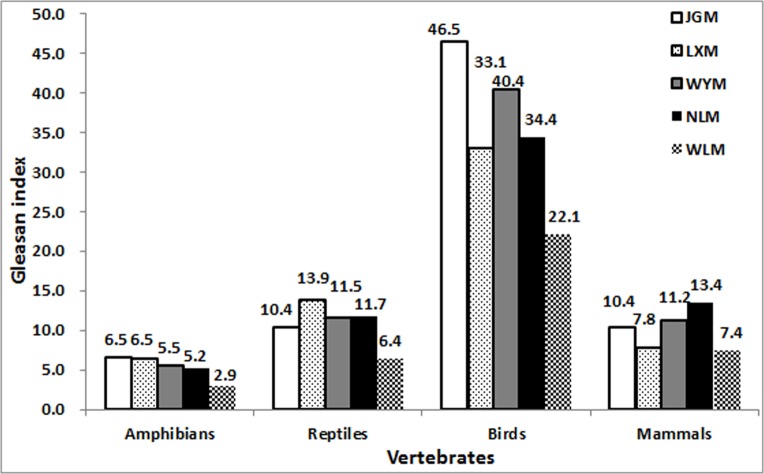
The Gleason index for animals in Jinggangshan Mountain (JGM). LXM, Luoxiao Mountain; WYM, Wuyi Mountain; NLM, Nanling Mountain; WLM, Wuling Mountain.

Compared to the surrounding mountains, the diversity of bird and amphibian species was higher on JGM (Table 4). The Gleason index for higher taxonomic levels was also higher for the three classes on JGM compared to the surrounding mountains (Fig. 3), especially for birds (Gleason index = 46.5). However, the abundance of mammalian species was relatively low. The Gleason index for animal species showed that the biodiversity on JGM was higher than on WLM, WYM, LXM, and NLM (Table 4).

**Table 4:** Comparison of the vertebrate biodiversity of JGM and surrounding mountains.

	Amphibian	Reptiles	Birds	Mammal	Sum	Area /km^2^	Gleason index
JGM	40	64	287	64	455	480	73.7
LXM	31	66	157	37	291	115	61.33
WYM	35	73	256	71	435	565.3	68.64
NLM	33	74	218	85	410	563	64.74
WLM	18	39	135	45	237	449	38.81

JGM, Jinggangshan Mountain; LXM, Luoxiao Mountain, WYM, Wuyi Mountain; NLM, Nanling Mountain; WLM, Wuling Mountain. The data for LXM, WYM, NLM and WLM came from surveys in Guanshan Natural Reserve [45], Wuyishan Area, Fujian Province [46], Nanling National Natural Reserve [55,56,57,58] and Badagongshan Mountain [59], respectively.

### Topographical characteristics

**Fig 4:**
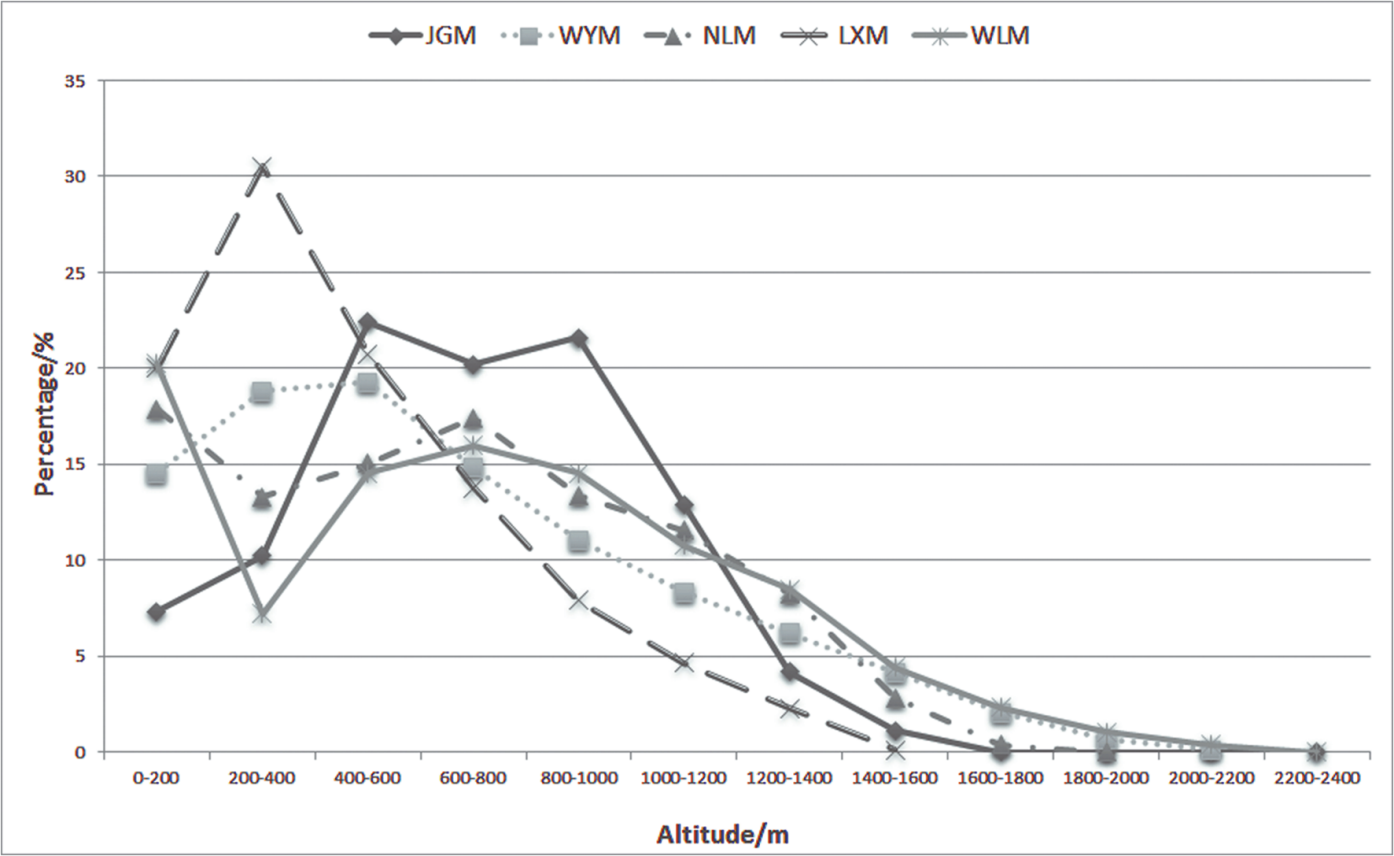
The percentage of altitudinal distribution of Jinggangshan Mountain (JGM) and surrounding mountains. LXM, Luoxiao Mountain; WYM, Wuyi Mountain; NLM, Nanling Mountain; WLM, Wuling Mountain.

More than 95% of the altitudinal distribution ranged between 0 m and 1600 m, and the mid-altitudes were considered to range from 400 m to 1200 m (Fig. 4). The mid-altitude region of JGM covered more than 77% of the mountain area, which is the highest value among the 5 mountains. The altitudes of the other 4 mountains mainly ranged from low to intermediate altitudes (from 200 m-1000 m).

**Fig 5:**
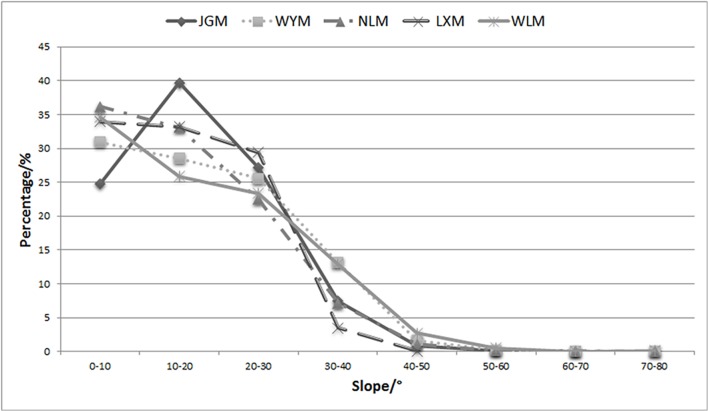
The percentage of slope distribution of Jinggangshan Mountain (JGM) and surrounding mountains. LXM, Luoxiao Mountain; WYM, Wuyi Mountain; NLM, Nanling Mountain; WLM, Wuling Mountain.

The slope proportion, ranging from 10°–20° in JGM, was higher than that of the other 4 mountains (Fig. 5).

### Overlapping species distribution

There were 8 animal species distributed in both JGM and LXM, and 7 species distributed in both JGM and NLM (Table 5). Both JGM and LXM belong to the Luoxiao Mountain range, and JGM and NLM were connected by a series of mountains without geographical isolation (Fig. 1), which may stimulate greater overlap of species (mainly frogs). There were 6 overlapping species (including 5 types of bird) between JGM and WYM. The long distance between may have led to only 1 species found in common between JGM and WLM.

**Table 5:** The distribution of overlapping animal species.

	JGM	LXM	WYM	WLM	NLM	Reference
*Megophrys jinggangensis*	√	√				[60]
*Megophrys lini*	√	√				[61]
*Megophrys cheni*	√	√				[61]
*Achalinus jinggangensis*	√	√				[62]
*Pachytriton archospotus*	√	√				[63]
*Latoucheornis siemsseni*	√		√			[64,65]
*Pteruthius xanthochlorus*	√		√			[64,65]
*Anthus roseatus*	√		√			[64,65]
*Sylviparus modestus*	√		√			[64,65]
*Coelops frithi*	√		√			[66,67]
*Quasipaa jiulongensis*	√		√			[68,69]
*Quasipaa boulengeri*	√			√		[68,69]
*Rana hanluica*	√	√			√	[68,69]
*Odorrana yizhangensis*	√				√	[68,69]
*Sphenomorphus tonkinensis*	√				√	[70]
*Phylloscopus yunnanensis*	√				√	[71]
*Megalaima oorti*	√	√			√	[64]
*Alcippe chrysotis*	√				√	[64]
*Dremomys pyrrhomerus*	√	√			√	[67]

√ stands for the species distributed in this mountain, and blank means the species didn’t find in this mountain.

There are more overlapping plant species distributed in JGM and WYM/WLM (166/192) than in JGM and LXM/NLM (71/67). The former two mountains are distributed to the east and west of JGM, and the latter two mountains are distributed to the north and south of JGM (Table 6). More overlapping plants species were distributed in JGM and the two mountains in the east (WYM) and west (WLM), while more overlapping animals species were distributed in JGM and the other two mountains in the north (LXM) and south (NLM).

**Table 6:** The distribution of overlapping plant species.

	No. of families	No. of genera	No. of species	No. of varietas
JGM+LXM	43	68	71	3
JGM+WYM	74	138	166	3
JGM+NLM	40	69	67	3
JGM+WLM	83	151	192	1

## Discussion

The species diversity in JGM area has been extensively studied (e.g. [72, 73, 74, 75]). However, most of them focus on specific groups of species. In the present study, a comprehensive survey on animals and plants was conducted in JGM area and the results were compared to those of the surrounding mountains. This study was among the first to explore the association between biodiversity and topography.

Topography plays an important role in biodiversity and community structure [23]. Mountains are important sources of water, energy, minerals, forest and agricultural products and areas of recreation, and they are storehouses of biological diversity, home to endangered species and an essential part of the global ecosystem [76]. Our results showed that JGM has overall higher biodiversity than the control areas. In total, there were 2958 spermatophyte species (belonging to 1003 genera and 210 families), and many endangered species were found in JGM (Table 3 and 4). In addition, the analysis of elevation and slope suggested that the geographic characteristics of JGM were unique among these mountains.

In a specific geographical region, the flora form depends on the heat, light, temperature, and humidity [77]. Previous studies mainly focused on climatic factors at large scales and demonstrated that the level of biodiversity in a region depends on climate [78]. Our results suggest that topographic conditions may also affect biodiversity. A previous study suggested that the latitudinal gradient of beta diversity could be partly explained by topography [79]. It has been shown that there are strong topoclimatic effects on butterfly diversity in a mountain range, and climate factors related to elevation appear to be the most important predictors of butterfly species richness on a fine scale [17]. One of the generally recognized patterns is a mid-altitude peak in species richness (midpeak) [38,39]. For example, Wilson et al. (2005) found unimodal curves for probability of occupancy against elevation. Higher native biodiversity or greater species density usually occurs at intermediate altitudes [17,26,28] where species have the highest probability of occupancy [30]. In the present study, the mid-altitude region of JGM covered more than 77% of the total mountain area, which was the highest proportion among the 5 studied mountains, while the altitudinal distribution of the other 4 mountains mainly ranged from low to intermediate altitude (from 200 m-1000 m). Previous studies on mountainous regions in south China also suggested that zonal vegetation distributed mostly in mid-altitude areas, while low-altitude areas usually suffered high level of human disturbance with mostly planted forest, and high-altitude areas usually supported less species because of the harsh climate conditions [80, 81].

In addition, the slope distribution of JGM ranged from 10°–20° and was greater than that of the other 4 mountains. Lower- and mid-range slopes are more suitable for the plant establishment and spread compared to steep slopes (>30°). High proportion of gentle slopes in JGM may be beneficial to species colonization and therefore boosting the formation of diverse communities. The higher biodiversity in JGM relative to its adjacent mountains may be due to its friendly altitude and slope.

Topography is an important factor affecting vegetation structure and species diversity by providing (micro-) habitat heterogeneity [82,83,84]. The habitat-heterogeneity hypothesis states that more complex habitats contain more niches and therefore higher species richness [15]. JGM has complex geography. There are 9 first-level habitats (including almost all the habitats except marine habitat) in JGM, suggesting the habitat type is plentiful [36]. There are also beaded basins (depressions) with dendritic water systems among the hills, and there are riffles and rapids in some regions, forming waterfalls in narrow gorges [37]. The variation of habitat types may result in the overall higher biodiversity in JGM than that of the surrounding “patches”.

Furthermore, vegetation complexity has been consistently recognized as one of the most important factors in supporting species richness and composition [16,85]. Chen et al. (2012) compared the vegetation types of JGM with adjacent heritage areas and found there were 90 vegetation formations and 180 associations in JGM which were substantially higher than that of other areas, while JGM is even in a smaller area (261.4 km^2^). By contract, there were only 34 vegetation formations in Wuyishan Mountain with an area of 565.27 km^2^ [36]. This vegetation complexity forms the base of a diverse community. Within a certain range, the biodiversity depends on the diversity of habitat and the key constructing elements. The great number of niches produced by various habitats in JGM may explain the accumulation of species in this area. Combining with our results, such high vegetation complexity might result from the unique topography characteristics, i.e. altitude and slope, and eventually lead to the support of higher biodiversity.

In addition, the geographic location of JGM may play a role in maintaining high biodiversity in this area. On one hand, JGM is surrounded by 4 other mountains (Fig. 1), which makes JGM like a central hub. Geographically, Wuyi Mountain blocks the eastern marine monsoon, and Wuling Mountain blocks the northwestern cold air, forming an enclosed region in JGM. Although we were unable to identify where all the species originate in these areas, the higher proportion of overlapping species suggested that JGM might be a more suitable surviving area for plants and animals than the adjacent mountains due to its unique topographic characteristics. On the other hand, biodiversity increasing within JGM at broad spatial scales may result from edge effects from the adjacent biomes. According to Wu [86] and “Physical Geography of China” [87], the flora of China can be partitioned into 2 floral regions, 7 floral subregions and 22 floral provinces. The floras of the four surrounding mountains belong to the same subregion, but different provinces. The flora of WLM belongs to the Central China floral province, while those of NLM and WYM belong to the South China floral province and the flora of LXM belongs to the East China floral province. Thus, the four mountains differ in their floral composition. The central zone, JGM, is surrounded by four large, heterogeneous “patches”. The unique topographic conditions of JGM provide stable topoclimate, which may partly explain the higher biodiversity on JGM.

The present study provided insights into the relationship between unique topography and biodiversity in the JGM area. Both microhabitat heterogeneity and vegetation complexity may contribute to sustain the high biodiversity in JGM. It should be noted that although the impact of topography on biodiversity was substantial, climate is still a more general factor influencing the formation and maintenance of greater biodiversity. Maintaining diversity across a varied topography might be a good way to conserve and improve biodiversity in a certain region.
